# Equitable implementation of lung cancer screening: avoiding its potential to mirror existing inequities among people who use tobacco

**DOI:** 10.1007/s10552-023-01790-z

**Published:** 2023-09-15

**Authors:** Emily Bilenduke, Shacoria Anderson, Alison Brenner, Jessica Currier, Jan M. Eberth, Jaron King, Stephanie R. Land, Betsy C. Risendal, Jackilen Shannon, Leeann N. Siegel, Mary Wangen, Austin R. Waters, Whitney E. Zahnd, Jamie L. Studts

**Affiliations:** 1 Department of Psychology, University of Colorado Denver, Denver, CO USA; 2 Department of Behavioral, Social, and Health Education Sciences, Rollins School of Public Health, Emory University, Atlanta, GA USA; 3 Division of General Medicine and Clinical Epidemiology, University of North Carolina School of Medicine, Chapel Hill, NC USA; 4 Lineberger Comprehensive Cancer Center, University of North Carolina, Chapel Hill, NC USA; 5 Center for Health Promotion and Disease Prevention, University of North Carolina, Chapel Hill, NC USA; 6 Division of Oncological Sciences, Knight Cancer Institute, Oregon Health and Science University, Portland, OR USA; 7 Department of Health Management and Policy, Drexel University, Philadelphia, PA USA; 8 Department of Epidemiology and Biostatistics, University of South Carolina, Columbia, SC USA; 9 Department of Health Promotion, Education, and Behavior, Arnold School of Public Health, University of South Carolina, Columbia, SC USA; 10 Tobacco Control Research Branch, Behavioral Research Program, Division of Cancer Control and Population Sciences, National Cancer Institute, Bethesda, MD USA; 11 Department of Community and Behavioral Health, Colorado School of Public Health, Cancer Prevention and Control Program, University of Colorado Cancer Center, Aurora, CO USA; 12 Department of Health Policy and Management, Gillings School of Global Public Health, University of North Carolina, Chapel Hill, NC USA; 13 Department of Health Management and Policy, College of Public Health, University of Iowa, Iowa City, IA USA; 14 Division of Medical Oncology, Department of Medicine, University of Colorado School of Medicine, Cancer Prevention and Control, University of Colorado Cancer Center, Aurora, CO USA

**Keywords:** Lung cancer screening, Equity, Tobacco use, Intersectionality, Community engagement

## Abstract

**Purpose:**

Lung cancer is the leading cause of cancer death, but the advent of lung cancer screening using low-dose computed tomography offers a tremendous opportunity to improve lung cancer outcomes. Unfortunately, implementation of lung cancer screening has been hampered by substantial barriers and remains suboptimal. Specifically, the commentary emphasizes the intersectionality of smoking history and several important sociodemographic characteristics and identities that should inform lung cancer screening outreach and engagement efforts, including socioeconomic considerations (e.g., health insurance status), racial and ethnic identity, LGBTQ + identity, mental health history, military experience/veteran status, and geographic residence in addressing specific community risk factors and future interventions in efforts to make strides toward equitable lung cancer screening.

**Methods:**

Members of the Equitable Implementation of Lung Cancer Screening Interest Group with the Cancer Prevention and Control Network (CPCRN) provide a critical commentary based on existing literature regarding smoking trends in the US and lung cancer screening uptake to propose opportunities to enhance implementation and support equitable distribution of the benefits of lung cancer screening.

**Conclusion:**

The present commentary utilizes information about historical trends in tobacco use to highlight opportunities for targeted outreach efforts to engage communities at high risk with information about the lung cancer screening opportunity. Future efforts toward equitable implementation of lung cancer screening should focus on multi-level implementation strategies that engage and work in concert with community partners to co-create approaches that leverage strengths and reduce barriers within specific communities to achieve the potential of lung cancer screening.

## Background

The lung cancer story in the United States (US) inevitably begins by acknowledging that lung cancer is the leading cause of cancer death among both men and women [1]. According to the American Cancer Society (ACS), approximately 238,290 Americans will be diagnosed and 127,070 will die from lung cancer in 2023, which is only slightly less than breast, colorectal, and prostate cancer combined [2]. The burden of lung cancer continues to decline due to innovations in lung cancer risk reduction, early detection, diagnosis, treatment, survivorship, and end-of-life care [1]. While this is no time to rest or claim victory, the growing optimism and hope surrounding lung cancer advances create an even greater potential to accelerate the trajectory of these benefits across the lung cancer continuum. However, implementation of these advances in risk reduction, early detection, and treatment has been hampered by substantial barriers across the structures and systems of the socio-ecological spectrum (e.g., healthcare system access, clinician practice changes, community awareness, and others).

The advent of evidence-based and policy-supported lung cancer screening for individuals at high risk for lung cancer constitutes one of the most substantial changes in the lung cancer landscape. The National Lung Screening Trial (NLST) results demonstrated a 20% relative reduction in lung cancer mortality and a nearly 7% decline in all-cause mortality associated with annual low-dose computed tomography (LDCT) in comparison to annual chest X-rays [3]. These data stimulated screening guideline development by all relevant authoritative organizations in the US and supported policy changes. The formulated guidelines consistently recommended lung cancer screening for those individuals who met the NLST eligibility criteria, based on age, pack-years, and in some cases, used expanded eligibility criteria [3]. For example, the US Preventive Services Task Force (USPSTF) guideline in late 2013 recommended lung cancer screening for individuals age 55 to 80 with at least a 30 pack-year history of smoking and who currently smoke or have quit within the last 15 years [4]. The expanded eligibility criteria, included in the 2021 update of the USPSTF guidelines, recommend to begin LDCT-based screening for people aged 50 to 80 years with a 20 pack-year history and those who currently smoke or have quit within the last 15 years [5, 6]. Because lung cancer screening utilizes this exposure-targeted approach based partly on smoking history, a highly stigmatized factor, implementation benefits stem from the ability and opportunity to identify risk and determine eligibility.

Like other cancer screening modalities, lung cancer screening is a process, beginning with the initial shared decision-making process through ongoing engagement with recommended follow-up scans and behavior change recommendations [7, 8]. Regrettably, efforts to offer lung cancer screening to the community have been challenging and slow. Current national data suggest that fewer than 10% of individuals who are eligible for lung cancer screening in the US (based on the original eligibility criteria) have been screened for lung cancer using the LDCT platform [9]. Other evidence-based and policy-supported screening programs for breast, colorectal, and cervical cancer achieve substantially higher levels of utilization, although they have been recommended longer [10]. Slow and potentially differential implementation of lung cancer screening, therefore, constitutes a significant health disparity.

The public health and tobacco control communities have developed a comprehensive understanding of smoking and tobacco use in the US using federal surveys (e.g., Behavioral Risk Factor Surveillance System) and other data addressing this key health metric. Utilizing that data, we can identify communities that are likely to experience higher rates of eligibility for lung cancer screening and evaluate lung cancer screening implementation in these communities to identify early indicators of disparity. Within these communities it is important to acknowledge that the disparity in screening experienced is a result of structures and systems intersecting to compound disadvantage and marginalization of communities that have been labeled for research purposes. While this commentary discusses known patterns of heightened smoking among specific communities, it also highlights unique and important opportunities to engage communities to co-design targeted lung cancer screening messaging and outreach. We propose that targeted and community-engaged messaging and outreach efforts in partnership with community representatives will be essential to achieving optimal and equitable lung cancer screening implementation and outcomes.

## Purpose

This commentary is a product of the Equitable Implementation of Lung Cancer Interest Group within the Cancer Prevention and Control Research Network (CPCRN) [11]. The CPCRN aims to accelerate uptake of evidence-based strategies in cancer prevention and control in communities, increase implementation and reach in underserved populations to reduce disparities, investigate determinants of implementation and programmatic success, and develop the workforce in cancer prevention and control research [11]. The Equitable Implementation of Lung Cancer Interest Group exemplifies the mission of the CPCRN by generating ideas and collaborations to develop an evidence base of implementation strategies which seek to eliminate disparities related to social drivers of health and address avoidable differences that hinder specific groups or communities from accessing the potential health benefits of lung cancer screening. This commentary is a call to action; highlighting the need to identify communities at greatest risk for lung cancer and with poorest access to primary care and lung cancer screening. These communities may benefit from targeted efforts to reduce systemic barriers and increase screening uptake. The information and solutions presented reflect the authors’ areas of expertise and the available literature.

## Current considerations

Intersectionality, a term first described by Crenshaw in 1989, is founded in critical theory and recognizes that inequities in society can overlap across various demographic groups, which has the effect of amplifying the social disparity identified in individuals with multiple minoritized identities [12]. While a full exploration of the vital components of an intersectional framework importantly consider structural and systemic factors (e.g., laws, policies, structural racism, societal smoking stigma, tobacco industry manipulations, and others) that interact with individual factors to exacerbate disadvantage, oppression, and marginalization, a comprehensive exposition of this important perspective is beyond the scope of this work. However, readers are encouraged to consider other work that provides a more extensive discussion of intersectionality, including works by Wilson and colleagues, which addresses intersectionality in clinical medicine [13] and by Turan and colleagues, which focuses on intersectional stigma and health [14].

Eligibility for lung cancer screening is largely driven by smoking history and there are well-documented community patterns in smoking that should be considered in light of substantial intersectionality. Disparities in tobacco use and tobacco cessation referrals may be early indicators of future differences in lung cancer screening uptake. For example, the combined effects of a person experiencing mental illness, low educational attainment, income difficulty, lesbian, gay, bisexual, transgender, queer, or any other identity other than heterosexual and cisgender (LGBTQ +) identity, and other considerations can overlap to create a significantly higher risk of smoking and tobacco use prevalence than any one identity alone [15–21]. These same groups may also experience lower levels of lung cancer screening. Research in lung cancer screening has recently begun to examine intersectionality as a lens to address community-level disparities [22–24] to create a unique opportunity to lean on decades of tobacco control work to facilitate equitable lung cancer screening.

The socio-ecological model (SEM) framed the exploration of the relationship between tobacco use and uptake of lung cancer screening, considering how individuals are affected by complex social influences nested within environmental interactions [25, 26]. For example, an individual who smokes may be influenced by the interpersonal relationships, organizations, communities, and policies with whom they interact; these influences may also impact their access to primary care and lung cancer screening. This commentary focused on the community level of the SEM by considering the potential for intersectional impact of several sociodemographic characteristics and identifying parameters on patterns of uptake of lung cancer screening, including health insurance, racial and ethnic identity, LGBTQ + identity, mental health, veteran status, and geographic residence.

*Health Insurance*. Health insurance and the ability to afford recommended preventative care is a major determinant of access to health care within a community. As noted above, the USPSTF recommendation released in 2013 was in favor of lung cancer screening; it was therefore covered as a preventive service by private insurance starting in 2015.[4] Subsequent favorable policy changes added coverage for Medicare beneficiaries in 2015 as well [27, 28]. With the updated USPSTF guidelines in 2021 and the expanded CMS coverage decision in 2022, insurance coverage was expanded to include the newly eligible population of individuals with lower smoking history and younger ages [5, 29]. However, coverage, co-pays, and other requirements (e.g., prior authorizations) by public and private payers vary by state/insurer, particularly involving Medicaid coverage [30]. According to the CDC, “Current tobacco product use prevalence is higher among adults who were uninsured (27.3%), enrolled in Medicaid (28.6%), or had some other public insurance (21.3%) compared to adults with private insurance (16.4%) or Medicare only (12.5%).”[31] These observations are especially concerning because many of the states with the highest prevalence of tobacco use do not cover lung cancer screening through Medicaid [32], and health insurance is likely to be a persistent challenge to achieving equitable implementation of lung cancer screening, particularly among those with public or no insurance [6].

*Race and Ethnic Considerations.* Rates of lung cancer screening remain low relative to the number of adults who meet eligibility guidelines, and this is particularly true for African American/Black and Hispanic individuals, as well as Asian women. In 2015, the percentage of eligible adults based on USPSTF 2013 guidelines who received lung cancer screening was 4.9% among non-Hispanic White persons, 1.7% among non-Hispanic Black persons, and 0.7% among Hispanic persons [33, 34]. In addition to disparate rates of uptake of lung cancer screening based on the initial eligibility criteria, concerns have been raised that the eligibility criteria may not appropriately account for lung cancer risk among non-White individuals. For example, African American/Black adults have a higher risk of lung cancer than non-Hispanic Whites, with fewer years of smoking [35], but are less likely to be eligible for screening based on current pack-year criteria [36]. A similar pattern emerges for Hispanic individuals and Asian women; they experience higher rates of lung cancer while often not meeting current pack-year eligibility criteria [37, 38]. Even with the 2021 update of the USPSTF recommendations to increase eligibility across all racial and ethnic groups by expanding eligibility, disparate eligibility remains for non-Hispanic Blacks and Asian women when evaluating relative risk compared to pack-year smoking history [37, 39].

*Sexual and Gender Minorities.* The rate at which individuals identify as LGBTQ + has grown substantially in the past decade, reaching 7.1% of the United States (US) population, over 23 million individuals, in 2022 [40, 41]. The LGBTQ + population includes individuals who hold an identity outside of the societal norm of heterosexual (i.e., opposite sex romantic or sexual attraction), cisgender (i.e., gender identity that matches recorded birth sex), and gender binary (i.e., the belief that there are only two genders, man and woman).

Disparities in smoking, tobacco use, and the associated negative consequences among LGBTQ + populations have been well documented for decades [42–45]. However, recent studies have identified sub-groups of the LGBTQ + community as particularly vulnerable to experimenting with tobacco products and transitioning to regular tobacco use. Specifically, young sexual minority groups, particularly lesbian and bisexual women have the highest prevalence of cigarette and e-cigarette use among all sexual minority and heterosexual counterparts [46, 47]. Structural stigma and discrimination in the form of laws, policies, and practices that result in unfair treatment of the LGBTQ + population further complicate smoking behaviors and is illustrated by a recent study indicating that transgender individuals who report structural discrimination were more likely to report smoking than those that do not report structural discrimination [48]. However, lung cancer screening rates remain similar to heterosexual counterparts, with the lowest cancer screening rates experienced by transgender individuals [49]. Low cancer screening rates more generally within the LGBTQ + population need to be contextualized in the vast literature surrounding healthcare-related discrimination [50].

*Mental Illness.* Compared to the general population, individuals experiencing mental illness have higher lung cancer incidence and mortality.[51] Higher cigarette smoking rates and exposure volume for people experiencing mental illness are the primary contributors [52]. Individuals with mental illness are twice as likely to smoke cigarettes and are less likely to access effective tobacco treatment programs to help them quit compared to individuals who do not have a mental illness [53, 54]. Additionally, individuals with mental illness may have lower rates of lung cancer screening that mirror the lower rates of engagement with tobacco cessation programs and lower rates of preventative screening for other cancers [55]. However, lung cancer screening access and uptake among individuals with mental illness remains understudied [55] and under-addressed clinically, creating the potential that lung cancer screening implementation will mirror disparities similar to the underutilization of tobacco cessation and other screenings among people experiencing mental illness.

*Military Veterans.* Tobacco use is more common among veterans compared to non-veterans age 50 years and younger [56]. As of 2015, approximately 21.6% of veterans reported current cigarette smoking and in 2018, almost 15% of veterans who were enrolled in healthcare reported current smoking [57]. Veterans are an important group to consider when addressing tobacco concerns and lung cancer screening opportunities, particularly since a large percentage began smoking after enlisting [57]. Identifying the impact of intersectionality within the veteran population is valuable as identifying as male, with no insurance access, low socioeconomic status, low education, and mental health concerns are associated with increased tobacco usage [56, 58, 59] and therefore increased likelihood to be eligible for lung cancer screening. Due to disparities in smoking rates among veterans and gender differences in smoking, there is a risk that similar groups that experience higher rates of smoking will also experience lower levels of lung cancer screening. It is imperative to continue to assess equitable lung cancer screening opportunities among this group, as veterans eligible for screening may be older, more likely to be male, and more likely to currently smoke compared to NLST participants [60].

*Geographic Residence.* Geography is an important factor to consider in the equitable provision of lung cancer screening as tobacco use and population distribution by age and subsequently, eligibility for lung cancer screening varies across regions and the rural–urban continuum [61, 62]. Lung cancer screening facilities are scarcer in areas of greatest risk [63–65], such as rural areas, where lung cancer mortality rates are higher. For example, one study found that over 17% of eligible rural persons had no access to a screening center within 40 miles, compared to less than 2% of urban persons [66]. Although geographic inequities in lung cancer screening access remain, the availability of screening locations has improved over time, providing greater opportunity for screening uptake. Lung cancer screening programs have been implemented in rural areas [67, 68], but additional interventions may be needed at the primary care level to ensure patients have the knowledge and resources to address specific barriers in order to engage in lung cancer screening [69, 70]. As rural individuals who are eligible for lung cancer screening are more likely to be current cigarette users [71] compared to urban patients eligible for screening, it is imperative for future efforts to ensure that patients have equitable geographic access to both screening- and evidence-based tobacco treatment interventions.

## Potential solutions

While there has been some focus on barriers to lung cancer screening at the community level of the SEM to promote lasting change, lung cancer screening considerations and modifications will need to be made on multiple levels (i.e., individual, interpersonal, organizational, and public policy) [72] For example, tobacco cessation efforts among veterans have included efforts to increase access to nicotine replacement therapy, elimination of outpatient copayment for smoking cessation counseling, and adoption of population-based approaches to smoking cessation [58] to address access, payment, and community barriers to cessation. Other strategies that have been used include a community-based “citizen scientist” approach and proactively mailed leaflets [73, 74] tailored to specific racial/ethnic minority populations and the creation of partnerships between radiology, primary care clinics, and mental health clinics to assist with shared decision-making counseling about lung cancer screening tailored for individuals with mental illness [75]. Patient navigation is another proven strategy for reducing structural barriers (e.g., transportation, insurance, scheduling, miscommunication) to care and is an evidence-based approach recommended in the CDC Community Guide to increase the uptake and delivery of breast, colon, and cervical cancer screening and could play a vital role in facilitating lung cancer screening [76]. Currently, there is some evidence for interventions to improve lung cancer screening uptake that are focused on reducing barriers for specific groups and for adapting previously established multi-level strategies that have been effective for other preventative cancer measures. Future efforts need to focus on adapting multi-level implementation strategies to improve lung cancer screening for people who may use tobacco or those who historically have been underutilizing LDCT screening. One ongoing effort leveraging multilevel interventions is working to facilitate colorectal cancer screening by delivering combined interventions addressing patient, provider, clinic, and community needs [77]. These strategies should not only engage multiple levels across the SEM, but they should also be informed by the full scope and depth of intersectionality theory, including the core tenets and principles to build coalitions, challenge current operations, and work toward achieving equity and justice with regard to the opportunity to participate in lung cancer screening.

Lung cancer screening interventions focused solely on the clinical system of delivery (i.e., targeting technology, capacity, expertise, and cost) [8] fail to fully consider the individuals and communities within which a lung cancer screening program resides. Community, whether defined as a shared geographic region, shared beliefs, or shared background, may play an outsized role in individual decision-making regarding participation in health care and attitudes toward novel technology. Communities, like individuals, are highly variable, with differing relationships to health behaviors, health care, and health care systems. But within these communities and subpopulations, such as those described earlier in this commentary, are unique assets and opportunities for collaboration and co-creation of messaging and outreach efforts around lung cancer screening with individuals embedded in the communities (community partners). Adapting implementation strategies using an asset-based approach, in collaboration with community partners, may be an effective model of contextualization, given the potential intersectionality of tobacco use history (a stigmatized condition) and community characteristics [78, 79]. For example, in a recent highly successful lung cancer screening implementation project, researchers used a community-engaged approach that incorporated a multidisciplinary group of partners, representing cancer clinicians, primary care, and supportive services to facilitate quality service delivery to co-develop the program strategies [67]. Because this work was carried out by a team of local clinicians and partners, they brought a deep knowledge of their community and challenges that would be unique to their community and patient population. The program was highly successful, resulting in a nearly five-fold increase in screening uptake over the course of the project [63].

## Conclusion

Lung cancer screening is fairly new and remains underutilized in all eligible groups and can therefore be considered a broad cancer screening disparity when compared against other cancer screening modalities. Unfortunately, there are early indicators suggesting that lung cancer screening is not yet reaching known at-risk communities. Building on the concept of intersectionality and extensive historical data describing smoking patterns in the US, there are notable opportunities to proactively engage communities based on sociodemographic characteristics or identifying attributes that are likely to experience disparities. Leveraging the knowledge of patterns of smoking and using community-engaged approaches through collaboration with individuals and organizations that represent these valued communities, the lung cancer screening community has the opportunity to develop and implement strategies that prevent, or at least minimize current and future inequity in lung cancer screening. Co-designing targeted messaging and outreach efforts along with modifying program operations in consideration of barriers to equitable access hold substantial potential, and these initiatives are urgently needed if the desired goal of equitable implementation of lung cancer screening is to be achieved.

## Data Availability

Data sharing not applicable to this article as no datasets were generated or analyzed during the current study.
